# Supplementary material for: A Comprehensive Investigation of Macro-Composition and Volatile Compounds in Spring-Picked and Autumn-Picked White Tea
Source: Foods. 2022 Nov 14;11(22):3628. doi: 10.3390/foods11223628 (PMC9688969; doi:10.3390/foods11223628)
Supplement: Supplementary file 1 [file foods-11-03628-s001.zip › Supplementary Figures.pdf]

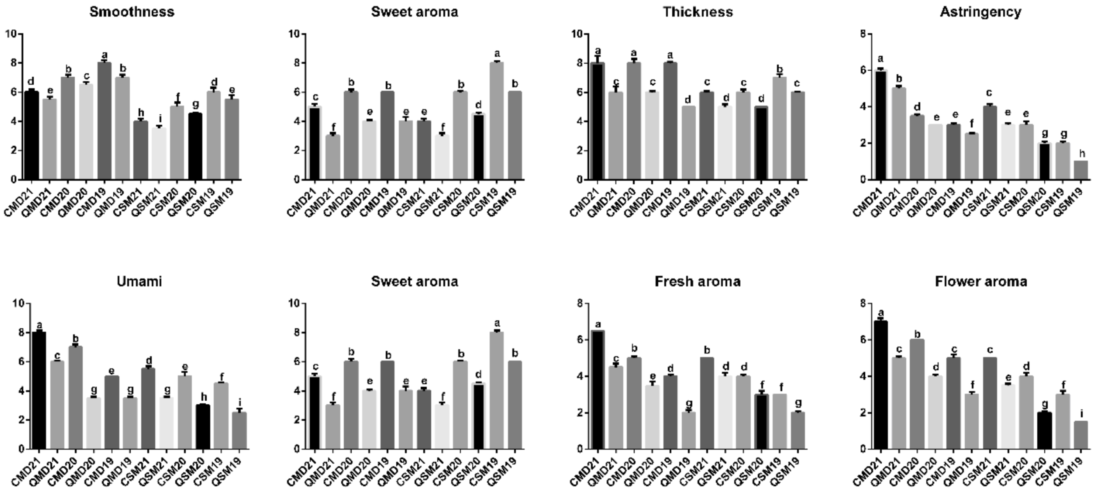

Figure S1. The results of the significant difference analysis of the taste and aroma attribute scores, The various superscripts show significant differences (p < 0.05).

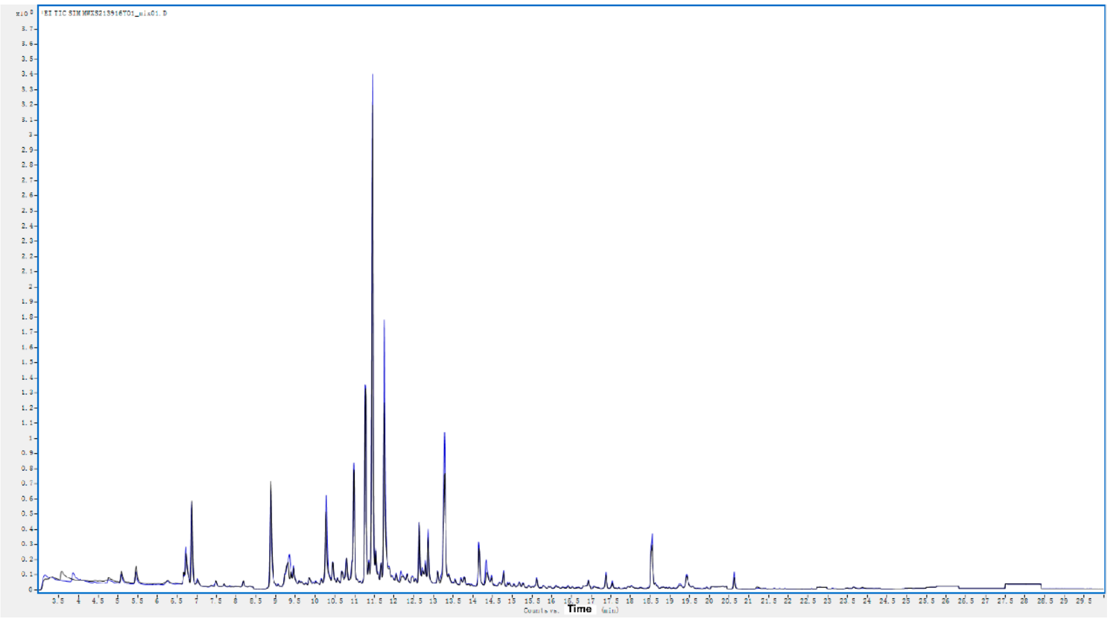

Figure S2. Total ion chromatogram of mixed sample mass spectrometry analysis for quality control

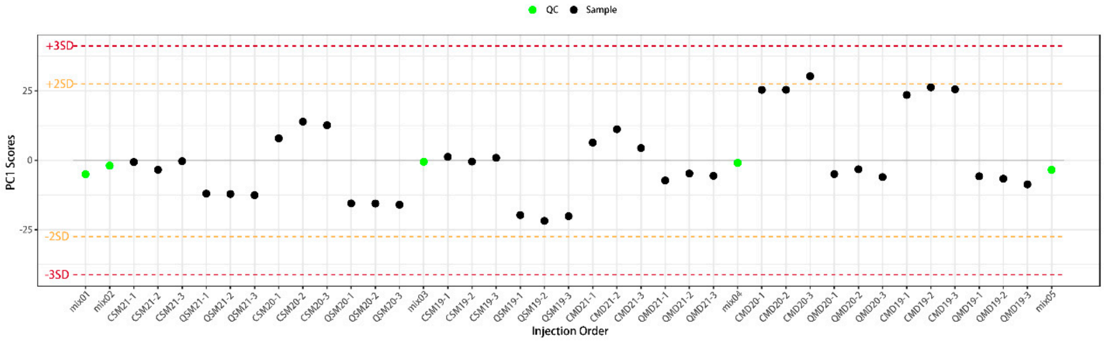

Figure S3: Overall sample PC1 control chart; Table S1: White tea sample information
